# Sensor Compromise Detection in Multiple-Target Tracking Systems

**DOI:** 10.3390/s18020638

**Published:** 2018-02-21

**Authors:** Juan-Pablo Ramirez-Paredes, Emily A. Doucette, Jess W. Curtis, Victor Ayala-Ramirez

**Affiliations:** 1 Department of Electronics Engineering, University of Guanajuato, Salamanca, Gto. 36885, Mexico; ayalav@ugto.mx; 2 Munitions Directorate, Air Force Research Laboratory, Eglin AFB, FL 32542, USA; emily.doucette@us.af.mil (E.A.D.); jess.curtis@us.af.mil (J.W.C.)

**Keywords:** sensor networks, estimation, cyberphysical systems

## Abstract

Tracking multiple targets using a single estimator is a problem that is commonly approached within a trusted framework. There are many weaknesses that an adversary can exploit if it gains control over the sensors. Because the number of targets that the estimator has to track is not known with anticipation, an adversary could cause a loss of information or a degradation in the tracking precision. Other concerns include the introduction of false targets, which would result in a waste of computational and material resources, depending on the application. In this work, we study the problem of detecting compromised or faulty sensors in a multiple-target tracker, starting with the single-sensor case and then considering the multiple-sensor scenario. We propose an algorithm to detect a variety of attacks in the multiple-sensor case, via the application of finite set statistics (FISST), one-class classifiers and hypothesis testing using nonparametric techniques.

## 1. Introduction

Including information from multiple cooperative sensors in a target tracking scenario can increase performance over using a single sensor or uncooperative sensors. For example, a bearings-only sensor requires more than one instance to recover point estimates of the target location. One concern that arises from the application of such a multiple-sensor system is its security. The sensors that comprise the system may come from various manufacturers and may be susceptible to interference by an adversary, either electronically or at their physical location. Therefore, as the number of sensors in a network increases, as does the number of potential vulnerabilities. This concern has been the subject of many studies by the cyberphysical systems community [1,2,3].

While there are a wide variety of attacks that a sensor network can suffer, one potential vulnerability comes from an adversary compromising some sensor nodes. An attack that completely disables some sensors can be easily detected, and thus appropriate measures can be taken to diminish its impact. Alternatively, an attacker could inject false information or a bias into the system so that its core algorithms fail. Detecting such false information is a challenge, because the specific nature of that kind of disruption is not known in advance.

The computing community has long studied the intrusion detection problem in the context of network security. Most work has consisted of analyzing the data traffic in search of unusual patterns [4,5,6]. In a wider context, the terms *anomaly* and *outlier* detection describe the search of any patterns that do not fit the nominal operation of a system. Searching for anomalies can be used for different purposes, such as for finding system faults or detecting intrusions [7,8].

In the context of a target tracking system, an attacker would be interested in disrupting the operation of the whole system to prevent some or all targets from being detected or to enable a tracked target to become unobservable or evade pursuit. A multiple-sensor system would be less vulnerable to a full takeover as a result of redundancy. As an alternative, an attacker in control of several nodes could introduce false measurements that deviate the tracker from the true target, making it follow a decoy or causing any estimation algorithms to diverge. Depending on the model, a simple disagreement among sensors may suffice to flag a possible compromise [9].

When the underlying system dynamics follow a linear time-invariant (LTI) model, it has been shown that it is possible to deal with additive disturbance signals, successfully recovering the system state [10,11,12]. If the specific nature of the attack cannot be anticipated, the search for anomalies in a system can be treated as a one-class classifier [13]. In this case, the nominal operation of the system has to be known beforehand, and a model of the measurements that it produces is built for later comparison. One of the earliest approaches to outlier detection consists of building a probabilistic model, such that inliers are considered to originate from a known distribution [14]. This idea has been extended to multi-dimensional datasets [15]. A problem of multiple-sensor systems, which is closely related to our motivation, is fault detection and isolation (FDI). This issue is often approached by deploying per-sensor strategies, which are combined in a decision-making stage. Some recent works utilize this technique, combined with a priori knowledge of the system dynamics or for a class of systems [16,17,18].

In this work, we present an algorithm to detect an intrusion in a multiple-sensor system. The algorithm works for a class of attacks that introduce a malicious signal at some sensor nodes with the purpose of deviating a target state estimator from the truth. We have applied the principles of hypothesis testing and the one-class classifier, which collects information about the system during nominal operation. This knowledge has then been applied to Bayesian networks (BNs) to perform an online detection of anomalous behavior in any subset of the sensors.

## 2. Background

### 2.1. Problem Statement

We consider a multiple-target tracking system, in which the dynamics of each target are assumed identical and are described by a model of the form

(1) $$x_{k+1}=f\left(x_{k}\right)+w_{k}$$

The tracking system collects observations from one or more sources, each following the model

(2) $$z_{k}=h\left(x_{k}\right)+\nu_{k}$$

In order to fully represent a multiple-target distribution, it is necessary to introduce the concept of a finite set. We consider some state space $E_{s}$ for the targets. The set of all finite subsets of $E_{s}$ is $F(E_{s})$. Then, the multiple-target state at some time instant *k*, assuming M(k) targets are present, is given by

(3) $$X_{k}=\left\{x_{k,1},\ldots ,x_{k,M(k)}\right\}\in F\left(E_{s}\right)$$

Similarly, $E_{o}$ is the space of all possible observations, and the N(k) multiple-target measurements at time *k* are given by

(4) $$Z_{k}=\left\{z_{k,1},\ldots ,z_{k,N(k)}\right\}\in F\left(E_{o}\right)$$

In a manner analogous to the way in which a vector can be a realization of a random process, a finite set is a realization of a random finite set (RFS). The class of RFSs that is considered in the derivation of the probability hypothesis density (PHD) filter is a Poisson point process. The RFS that represents the multiple-target state at time *k* is

(5) $$\Xi_{k}=S_{k}\left(X_{k-1}\right)\cup B_{k}\left(X_{k-1}\right)\cup \Gamma_{k}$$

where $S_{k}\left(X_{k-1}\right)$ is the RFS of the targets $X_{k-1}$ that survived to time *k*, $B_{k}\left(X_{k-1}\right)$ is the RFS of targets born from $X_{k-1}$, and $\Gamma_{k}$ is the RFS of targets spawned at time *k*.

As for the observations, their RFS at time *k* is

(6) $$\Sigma_{k}=\Theta_{k}\left(X_{k}\right)\cup K_{k}\left(X_{k}\right)$$

where $\Theta_{k}\left(X_{k}\right)$ is the RFS of measurements derived from the multiple-target state $X_{k}$, and $K_{k}\left(X_{k}\right)$ is the RFS of observations not related to targets, also known as clutter.

### 2.2. Multiple-Target Estimation with the PHD Filter

The PHD filter is a solution to the multiple-target tracking problem based on finite set statistics (FISST) to propagate a moment of the full multiple-target probability density function. This solution reduces the computational complexity while still allowing the model to accommodate for target births and deaths and false positive detections, that is, sensor clutter. A complete derivation of the PHD filter is given in [19], while details about its sequential Monte Carlo (SMC) implementation can be found in [20]. We will mention some of the basic concepts behind the PHD filter in order to introduce this estimator. These concepts are used later in this text to explain our treatment of the sensor compromise detection problem.

While there exists a set of sequential Bayesian inference equations to propagate the multiple-target distribution, there is a very high computational cost associated to them, making any practical implementations unfeasible. The PHD filter does not propagate the full multiple-target distribution.

It has been demonstrated in [19] that the PHD is the first moment of an RFS; thus (omitting time indices),

(7) $$\int_{S}D\left(x\right)dx=E\left[|\Xi \cap S|\right]$$

Similarly to sequential Bayesian inference, the PHD is a density that can be propagated using a prediction-update procedure. The PHD is defined in state space, instead of as a set-valued function. Thus, x and w in the following equations refer to points in the state space of the possible targets. The PHD prediction is given by

(8) $$\begin{array}{c} D_{k+1|k}\left(x\right)=b_{k+1|k}\left(x\right)+\\ \int \left(p_{S}\left(w\right)f_{k+1|k}\left(x|w\right)+b_{k+1|k}\left(x|w\right)\right)D_{k|k}\left(w\right)dw \end{array}$$

with $b_{k+1|k}\left(x\right)$ as the target birth intensity function at location x, $b_{k+1|k}\left(x|w\right)$ as the intensity function of targets spawned from w, $p_{S}\left(w\right)$ as the probability that a target at w still exists at k+1, and $f_{k+1|k}\left(\cdot |\cdot \right)$ as the transition probability density of individual targets.

The predicted PHD is then corrected using observations from the sensor in the update stage. First, we define the “pseudo-likelihood” term, given by

(9) $$F_{k+1}\left(Z_{k+1}|x\right)=1-p_{D}\left(x\right)+\sum_{z\in Z}\frac{p_{D}\left(x\right)L_{z}\left(x\right)}{\lambda c\left(z\right)+\langle D_{k+1|k},p_{D}L_{z}\rangle }$$

where c(z) is the clutter density function, the parameter λ is the average number of Poisson-distributed false alarms per sensor observation, and $P_{D}\left(x\right)$ is the detection probability. The function $L_{z}\left(z\right)=g\left(z|x\right)$ is the measurement likelihood function. Finally, 〈f,h〉=∫f(x)h(x)dx. Using $F_{k+1}\left(\cdot \right)$, the PHD update equation is

(10) $$D_{k+1|k+1}=F_{k+1}\left(Z_{k+1}|x\right)D_{k+1|k}$$

### 2.3. Multiple-Sensor PHD Filter

The problem of multiple-target tracking can have an increased complexity if several sensors are used at the same time, as noted in [19]. There is a high computational cost associated with exact multiple-sensor processing. In particular, the order in which their observations are processed can influence the outcome of the estimator. A simplification for sensors with non-overlapping fields of view or coverage regions is described in [21], while [22] and [23] propose alternative methods to process information from multiple sensors. The simplest approximation is the “pseudo-sensor”, which processes all of the observations as coming from a single source [24] but that has been shown to be inaccurate.

An approximate solution to the multiple-sensor processing problem that presents a reasonable compromise between accuracy and ease of implementation is the iterated-corrector [19]. We can define a modified Equation (9) to accommodate *s* different sensors as

(11) $$\begin{array}{cc} F_{k+1}^{\left[i\right]}\left(Z_{k+1}^{\left[i\right]}|x\right)= & 1-p_{D}^{\left[i\right]}\left(x\right)+\\ & \sum_{z\in Z^{\left[i\right]}}\frac{p_{D}^{\left[i\right]}\left(x\right)L_{z}^{\left[i\right]}\left(x\right)}{\lambda^{\left[i\right]}c^{\left[i\right]}\left(z\right)+\langle D_{k+1|k}^{\left[i-1\right]},p_{D}^{\left[i\right]}L_{z}^{\left[i\right]}\rangle } \end{array}$$

where $D_{k+1|k}^{\left[0\right]}\left(x\right)=D_{k+1|k}\left(x\right)$, and

(12) $$D_{k+1|k}^{\left[i\right]}\left(x\right)=F_{k+1}^{\left[i\right]}\left(Z_{k+1}^{\left[i\right]}|x\right)D_{k+1|k}^{\left[i-1\right]}\left(x\right)$$

Finally, the multiple-sensor PHD update is given by

(13) $$D_{k+1|k+1}\left(x\right)=F_{k+1}^{\left[s\right]}\left(Z_{k+1}^{\left[s\right]}|x\right)$$

### 2.4. Target Detection from the SMC–PHD Algorithm

The PHD is not a probability density, and moreover the PHD filter does not build target tracks on its own. In the case of the SMC implementation of the PHD filter, the PHD is encoded as a set of particles that represent different realizations of feasible targets over $E_{s}$. Each particle $x_{k}^{\left(i\right)}$ has an associated weight $w_{k}^{\left(i\right)}$. The number of particles is not normally constant over time; it changes with $L_{k}$, denoting how many particles are being used at time *k*.

In the SMC–PHD implementation, it is possible to obtain approximate target locations from the PHD with a clustering algorithm such as *K*-means [25] or *K*-medoids [26] acting on all $x_{k}^{\left(i\right)},\;i=1,\ldots ,L_{k}$. These clustering algorithms require the number of cluster centers to be specified in advance, and it can be obtained from the SMC–PHD as

(14) $$k=\left[{\hat{N}}_{k|k}\right]=\left[\sum_{j=1}^{L_{k-1}+J_{k}}w_{k}^{\left(j\right)}\right]$$

where [·] denotes rounding to the nearest integer.

The SMC–PHD algorithm has a resampling step at the end of each iteration, after which all of the particle weights are equal. Hence, the clustering algorithm operates only in the target state space with no consideration of ${\left\{w_{k}^{\left(i\right)}\right\}}_{i=1}^{L_{k}}$. We refer to the resulting finite set as ${\hat{X}}_{k}$.

## 3. Methods

### 3.1. Preliminaries

We assume that some region $E_{s}$ is being surveyed by *s* sensors. We can pose the sensor compromise problem as an anomaly detection application. Referring back to the RFS formulation of the observation set in Equation (6), the observation set with added anomalies for sensor *i* can be described as

(15) $${\tilde{\Sigma }}_{k}^{\left[i\right]}={\tilde{\Theta }}_{k}^{\left[i\right]}\left(X_{k}\right)\cup K_{k}^{\left[i\right]}\left(X_{k}\right)\cup \Delta_{k}^{\left[i\right]}$$

while in the nominal operation mode, the observation RFS $\Theta_{k}^{\left[i\right]}\left(X_{k}\right)$ follows a certain model that includes noise derived from a known distribution. In ${\tilde{\Sigma }}_{k}^{\left[i\right]}$, the observation ${\tilde{\Theta }}_{k}^{\left[i\right]}\left(X_{k}\right)$ has noise distributions that may not match the assumed model. The clutter model can also be modified by the attack. In addition, any attacks that consist of the introduction of decoys among the targets can be modeled as a new RFS, $\Delta_{k}^{\left[i\right]}$.

While it may not be possible to characterize each and every attack or sensor failure, the model used in Equation (15) allows us to enumerate the major categories into which an anomalous behavior may fall:

1. Modified observation of a given target: The sensor noise does not conform to the assumed distribution. This can be, for example, an added bias on the observations or an increased covariance in the noise distribution.
2. Modified probability of target detection: For instance, some targets can be omitted from the sensor observations.
3. Modified false alarm rate: This can include an increase in the clutter intensity, duplicate reports for a given target, and so on.

An attack or malfunction may combine any of the above.

**Figure 1:**
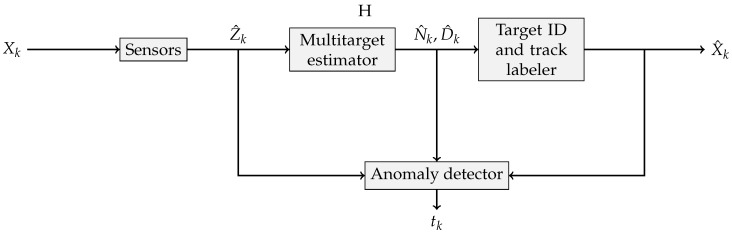
Anomaly detection scheme used in this work.

Confronted with the impossibility of modeling every kind of attack on the sensor system, an alternative is to resort to the one-class classifier approach. A diagnostics system is built that is only trained to recognize the nominal operation of the sensor array. Any measurements that deviate significantly from the learned model are then classified as being caused by a breach in the system or a hardware failure. The overall architecture of such an anomaly detection system is shown in Figure 1.

Selecting which features to learn about a multiple-target tracker is not straightforward. Because we do not limit our analysis to a specific class of dynamical systems or observation models, we inspire our anomaly detection approach in the concept of hypothesis testing.

The multiple-target scenario includes the possibility of having target births, deaths and sensor clutter. Hence, it requires a way to quantify the deviation from each sensor with respect to the estimator as a whole. Before continuing with our exposition, we introduce some assumptions that we require to hold for the anomaly detection techniques studied.

**Assumption** **1.** The observation spaces for all sensors are such that $E_{o}^{\left[i\right]}=E_{o}^{\left[j\right]}$ for i,j=1,…,s, or there exist one-to-one transformations $T_{i}^{j}$ such that $E_{o}^{\left[j\right]}=T_{i}^{j}\left[E_{o}^{\left[i\right]}\right]$.

**Assumption** **2.** $E_{o}$ is a Banach space, with metric d(x,y).

**Assumption** **3.** The observation noise for the sensor, with respect to each target, is a stationary process, with known mean. Moreover, the noise statistics for all observations from targets are equal and known.

The first assumption allows for the direct comparison of observations stemming from different sensors. The second enables us to use a metric to compare sensor observations. Finally, the third simplifies the analysis of the relative error between observations from different sensors.

### 3.2. OSPA Metric

For every estimation algorithm, there is a metric that can quantify its performance. As an example, any single-target estimation algorithm can use the $L_{2}$-norm to compute the difference between the estimated state and the true target state. This is no longer valid in the case of multiple targets, as the estimation error can consist not only of a distance in state space, but also of association errors between estimates and different target states. There can also be errors in the estimation of the cardinality of the target set. Metrics such as the Haussdorf distance have been proposed to capture these errors, and some developments have dealt with the limitations of this distance [27]. An alternative that solves most of these problems and remains a valid metric, that is, it satisfies the axioms of identity, symmetry and the triangle inequality, is optimal sub-pattern assignment (OSPA) [28]. To use this metric, one needs to specify a parameter called the cut-off c>0, in addition to 1≤p<∞, which is the order of OSPA.

The OSPA metric is computed using the expression

(16) $${\bar{d}}_{p}^{\left(c\right)}\left(X,Y\right)={\left(\frac{1}{n}\left(\min_{\pi \in \Pi_{n}}\sum_{i=1}^{m}d^{\left(c\right)}{\left(x_{i},y_{\pi (i)}\right)}^{p}+c^{p}\left(n-m\right)\right)\right)}^{1/p}$$

where $d^{\left(c\right)}\left(x,y\right)=\min\left(c,d\left(x,y\right)\right)$, and $\Pi_{k}$ is the set of permutations on {1,…,k} for any k∈N. The cardinalities of the sets being compared are |X|=m and |Y|=n. Equation (16) is only valid if m≤n; otherwise ${\bar{d}}_{p}^{\left(c\right)}\left(X,Y\right):={\bar{d}}_{p}^{\left(c\right)}\left(Y,X\right)$.

In the rest of this work, we make use of a decomposition of the OSPA metric into two components: localization error and cardinality error. The localization component is given by

(17) $$e_{p,\mathrm{loc}}^{\left(c\right)}\left(X,Y\right)={\left(\min_{\pi \in \Pi_{n}}\frac{1}{n}\sum_{i=1}^{n}d^{\left(c\right)}{\left(x_{i},y_{\pi (i)}\right)}^{p}\right)}^{1/p}$$

and the cardinality component is

(18) $$e_{p,\mathrm{card}}^{\left(c\right)}\left(X,Y\right)={\left(\frac{c^{p}\left(n-m\right)}{n}\right)}^{1/p}$$

As before, these expressions only hold valid if m≤n, and by definition, $e_{p,\mathrm{loc}}^{\left(c\right)}\left(X,Y\right):=e_{p,\mathrm{loc}}^{\left(c\right)}\left(Y,X\right)$ and $e_{p,\mathrm{card}}^{\left(c\right)}\left(X,Y\right):=e_{p,\mathrm{card}}^{\left(c\right)}\left(Y,X\right)$ if m>n.

### 3.3. Anomaly Detection: Single-Sensor Case

In the single-sensor case, the information available to the filter is limited to the estimated target states over time and the observations from a single source. Detecting an anomaly in the sensor behavior implies comparing its observations of the projections of the estimated states to $E_{o}$.

In the single-sensor, single-target case, one solution to identify whether an estimator is operating nominally is the use of residual analysis [29]. In a Kalman filter, the residual is given by $|y-C\hat{x}|$, where *y* is an observation and $\hat{x}$ is the estimated target state. The residual in this case follows a $\chi^{2}$ distribution; thus a hypothesis test would reveal the existence of an anomaly.

The single-sensor, multiple-target case with general nonlinear dynamics and non-Gaussian noise requires a different treatment. Each pair of dynamical and observation models would require a separate derivation of the statistics of the residuals. A general approach needs a training phase in which a statistical model of the residuals is built, to be used as the basis for anomaly detection during a deployment phase.

In a single-sensor, multiple-target tracker, the ability of an attacker to introduce decoys is limited by $b_{k+1|k}\left(x\right)$. In a trusted sensors framework, the target birth intensity is typically chosen to minimize the response time of the PHD filter, such that new targets are tracked as soon as possible. However, this renders any decoy injection attacks quite effective, as they all become new objects in the tracker. Tuning $b_{k+1|k}\left(x\right)$ can delay the apparition of any decoys and enable the detection of an attack by imposing a limit on the expected number of new targets, but at the expense of delaying the detection of true targets. Another problem is that this countermeasure assumes that the attacker does not know about the modified $b_{k+1|k}\left(x\right)$, as otherwise, they would just introduce the decoys sequentially over time to avoid detection.

**Proposition** **1.** Consider a single-sensor, multiple-target tracker based on the PHD filter. If an attacker has full knowledge of $b_{k+1|k}\left(x\right)$ and the expected number of targets to track is always unknown a priori, the attacker can introduce decoys without being detected.

**Proof.**  We show that even for the simplest multiple-target tracking case, an attacker would be capable of spawning a new target in the system. We consider a basic PHD filter with $p_{S}\left(x\right)=1$, $b_{k+1|k}\left(x|w\right)=0$ and a single target, such that $D_{k|k}\left(x\right)=f\left(x\right)$ is some unimodal density. The sensor is assumed to produce no clutter. Then,

$$D_{k+1|k}\left(x\right)=f_{1}\left(x\right)+b_{k+1|k}\left(x\right)$$

after the prediction stage. An observation of the true target $z_{1}$ is reported by the sensor. We assume that an attacker sends $z_{2}$, a false report of the location of a second target. For illustration purposes, we consider $L_{z_{1}}\left(x\right)$ and $L_{z_{2}}\left(x\right)$ to have negligible overlap. From Equation (9), it follows that each observation will produce a term in the sum to compute $F_{k+1}\left(Z_{k+1}|x\right)$. The term due to the real observation is

$$\frac{L_{z_{1}}\left(x\right)f_{1}\left(x\right)+L_{z_{1}}\left(x\right)b_{k+1|k}\left(x\right)}{\langle f_{1},L_{z_{1}}\rangle +\langle b_{k+1|k},L_{z_{1}}\rangle }$$

while the term corresponding to the decoy is

$$\frac{\overset{\to 0}{\overset{︷}{L_{z_{2}}\left(x\right)f_{1}\left(x\right)}}+L_{z_{2}}\left(x\right)b_{k+1|k}\left(x\right)}{\underset{\to 0}{\underset{︸}{\langle f_{1},L_{z_{2}}\rangle }}+\langle b_{k+1|k},L_{z_{2}}\rangle }\approx \frac{L_{z_{2}}\left(x\right)b_{k+1|k}\left(x\right)}{\langle b_{k+1|k},L_{z_{2}}\rangle }$$

and as a result of this reduction, integrating the resulting PHD produces

$$\int D_{k+1|k+1}\left(w\right)dw\approx N_{k+1|k+1}+1$$

The introduction of the extra decoy can only be prevented if $L_{z_{2}}\left(x\right)b_{k+1|k}\left(x\right)\approx 0\;\forall \;x$.

As we have shown, even under the simplest conditions, it would not be possible to detect the insertion of a decoy by an attacker. As those conditions are relaxed, and because clutter, splitting and overlapping targets are included in the model, there are even more possible locations in which an attacker can successfully place a decoy without violating the sensor model. ☐

### 3.4. Anomaly Detection for Multiple-Sensor Systems

As discussed so far, the detection of anomalies in the single-sensor case, for a multiple-target tracking problem with nonlinear dynamics, has several difficulties that may not have a viable solution for all scenarios. If a redundant architecture with multiple sensors is introduced instead, new possibilities arise for compromise detection. For the multiple-sensor case, it is possible to use the observations from all sensors to measure the deviation of each from a consensus, under the following condition.

**Assumption** **4.** An attacker cannot control more than $\tilde{s}\leq \left\lceil s/2-1\right\rceil$ sensors; thus a majority consensus set $Z_{k}$ with all observations by all sensors at time k can be constructed. From Assumption 1, every element of $Z_{k}$ belongs to a single $E_{o}$.

As established in the previous section, the OSPA metric can indicate distances between finite sets with different cardinalities. Hence, we propose using quantities related to the OSPA metric to measure the distance between the observations of a sensor, $Z^{\left[i\right]}$, and the estimated target states $\hat{X}$. Because OSPA combines localization and cardinality errors in a single metric, we opt to use $e_{p,\mathrm{loc}}^{\left(c\right)}\left(X,Y\right)$ and $e_{p,\mathrm{card}}^{\left(c\right)}\left(X,Y\right)$ as a way to separate the two kinds of sensing errors in multiple-target scenarios. Our anomaly detection goal does not need the symmetry property of OSPA and can benefit from a distinction between localization and cardinality disparities among sensors.

We consider, for each sensor *i*, the set of its observations $Z_{k}^{\left[i\right]}$. Hence, we can construct the set of all observations with the exception of those from sensor *i*, which are $Z_{k}\backslash Z_{k}^{\left[i\right]}$. In our exposition of the anomaly detection scheme, the *p* and *c* values of $e_{p,\mathrm{loc}}^{\left(c\right)}\left(X,Y\right)$ and $e_{p,\mathrm{card}}^{\left(c\right)}\left(X,Y\right)$ are assumed to remain constant; thus we do not refer to them explicitly unless necessary. A calculation of the localization and cardinality errors of the observations from each sensor *i* at time *k*, when compared to the consensus set, can be denoted by

(19) $$\begin{array}{c} \Lambda_{k}^{\left[i\right]}=e_{p,\mathrm{loc}}^{\left(c\right)}\left(Z_{k}^{\left[i\right]},Z_{k}\backslash Z_{k}^{\left[i\right]}\right) \end{array}$$

(20) $$\begin{array}{c} \Upsilon_{k}^{\left[i\right]}=e_{p,\mathrm{card}}^{\left(c\right)}\left(Z_{k}^{\left[i\right]},Z_{k}\backslash Z_{k}^{\left[i\right]}\right) \end{array}$$

Because the probability distributions of $\Lambda_{k}^{\left[i\right]}$ and $\Upsilon_{k}^{\left[i\right]}$ depend on many factors, such as the target dynamics, sensor models and number of targets, they can be constructed from experimental data during nominal system operation. A training phase with fully trusted sensors and estimator would be needed.

After the training data is gathered, sequences ${\bar{\Lambda }}_{k}^{\left[i\right]}=\Lambda_{k-l}^{\left[i\right]},\ldots ,\Lambda_{k}^{\left[i\right]}$ and ${\bar{\Upsilon }}_{k}^{\left[i\right]}=\Upsilon_{k-l}^{\left[i\right]},\ldots ,\Upsilon_{k}^{\left[i\right]}$ need to be analyzed for each sensor. An adequate hypothesis test is necessary to evaluate if the null hypothesis, $H_{0}:{\bar{\Lambda }}_{k}^{\left[i\right]},{\bar{\Upsilon }}_{k}^{\left[i\right]}∼p\left(\Lambda_{k}^{\left[i\right]},\Upsilon_{k}^{\left[i\right]}\right)$, can be rejected. Useful tools in this context are the Kolomogorov–Smirnov two-sample test, and the Wilcoxon rank-sum test [30,31]. For this work, we apply the latter.

There can be a coupling between the sensor outputs and the estimated state. It would be desirable to isolate a faulty sensor from others. Unfortunately, as shown by Equation (11), the iterated-corrector scheme for multiple-sensor information fusion processes the observations from each source in a sequential manner. Because we are proposing to compare each $Z^{\left[i\right]}$ to $\hat{X}$, and because $\hat{X}$ is extracted from $D_{k+1|k+1}\left(x\right)$, several sensors could be considered as anomalous, as $H_{0}$ is rejected for each of them. The nature of the attack determines whether faulty sensors can be isolated or not, as evidenced later in our results section.

### 3.5. Algorithm for Multiple-Sensor Anomaly Detection

We summarize our anomaly detection methodology. We base the anomaly detection procedure on a BN. The rationale behind this choice is the observation that the two measurements Λ and Υ may have different values depending on the class of the attack. Most attacks, if they are of high enough intensity, will cause a mismatch in the number of objects detected by a sensor. Only a subset of attacks that introduce a small error signal, such as noise or bias, will provoke an increase in Λ without affecting Υ.

**Figure 2:**
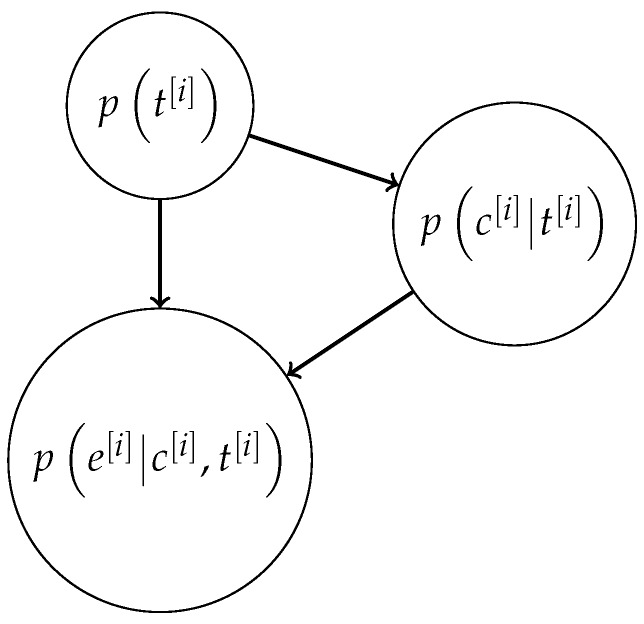
Bayesian network architecture for anomaly detection, for a single sensor.

1. *Data collection*. Gather data from all sensors during nominal operation. This requires a trusted environment. This data is then used to compute $\Lambda_{k}^{\left[i\right]}$ and $\Upsilon_{k}^{\left[i\right]}$ for all *k* in the recorded measurements.
2. *Nonparametric statistical test*. During a target tracking task, keep a history of measurements for every sensor using a fixed horizon; that is, collect ${\bar{\Lambda }}_{k}^{\left[i\right]},{\bar{\Upsilon }}_{k}^{\left[i\right]}$ for a lag *l*. At every time step *k*, perform a nonparametric statistical test between the history of measurements and the trusted data for each sensor, and save the *p*-value of the test.
3. *Bayesian inference for anomaly detection*. Denote the trustworthiness of each sensor by the indicator function:
  (21) $$t_{k}^{\left[i\right]}=\left\{\begin{array}{cc} 0 & \mathrm{Sensor}\;i\;\mathrm{cannot}\;\mathrm{be}\;\mathrm{trusted}\;\mathrm{at}\;\mathrm{time}\;k\\ 1 & \mathrm{Sensor}\;i\;\mathrm{is}\;\mathrm{fully}\;\mathrm{reliable}\;\mathrm{at}\;\mathrm{time}\;k \end{array}\right.$$
  There are two events associated with the result of a hypothesis test between a fixed number of observations of Λ and Υ:
  (22) $$c_{k}^{\left[i\right]}=\left\{\begin{array}{cc} 0 & \mathrm{Sensor}\;i\;\mathrm{reports}\;\mathrm{an}\;\mathrm{anomalous}\;\Upsilon \;\mathrm{at}\;\mathrm{time}\;k\\ 1 & \mathrm{Sensor}\;i\;\mathrm{has}\;a\;\mathrm{nominal}\;\Upsilon_{k} \end{array}\right.$$
  (23) $$e_{k}^{\left[i\right]}=\left\{\begin{array}{cc} 0 & \mathrm{Sensor}\;i\;\mathrm{reports}\;\mathrm{an}\;\mathrm{anomalous}\;\Lambda \;\mathrm{at}\;\mathrm{time}\;k\\ 1 & \mathrm{Sensor}\;i\;\mathrm{has}\;a\;\mathrm{nominal}\;\Lambda_{k} \end{array}\right.$$
  The results of the nonparametric hypothesis test provide $p(c_{k}^{\left[i\right]}|t_{k}^{\left[i\right]}=1)$ and $p(e_{k}^{\left[i\right]}|t_{k}^{\left[i\right]}=1)$. Then the probability of $t^{\left[i\right]}$ can be updated over time in a Bayesian manner. The inspection of the effects of some attacks on Λ,Υ reveals that the dependence relations among $t_{k}^{\left[i\right]}$, $c_{k}^{\left[i\right]}$ and $e_{k}^{\left[i\right]}$ can be described by the BN depicted in Figure 2. Hence, the posterior probability for $t^{\left[i\right]}$ is given by
  (24) $$\begin{array}{c} p\left(t_{k}^{\left[i\right]}|e_{k-1}^{\left[i\right]},c_{k-1}^{\left[i\right]},t_{k-1}^{\left[i\right]}\right)=\sum_{j}f\left(t_{k}^{\left[i\right]}|t_{k-1}^{\left[i\right]}=j\right)p\left(t_{k-1}^{\left[i\right]}=j|e_{1:k-1}^{\left[i\right]},c_{1:k-1}^{\left[i\right]}\right) \end{array}$$
  (25) $$\begin{array}{c} p\left(t_{k}^{\left[i\right]}|e_{k}^{\left[i\right]},c_{k}^{\left[i\right]}\right)=\frac{p\left(e_{k}^{\left[i\right]}|t_{k}^{\left[i\right]}\right)p\left(c_{k}^{\left[i\right]}|e_{k}^{\left[i\right]},t_{k}^{\left[i\right]}\right)p\left(t_{k}^{\left[i\right]}|e_{k-1}^{\left[i\right]},c_{k-1}^{\left[i\right]},t_{k-1}^{\left[i\right]}\right)}{\sum_{j}p\left(e_{k}^{\left[i\right]}|t_{k}^{\left[i\right]}=j\right)p\left(c_{k}^{\left[i\right]}|e_{k}^{\left[i\right]},t_{k}^{\left[i\right]}=j\right)p\left(t_{k}^{\left[i\right]}=j|e_{k-1}^{\left[i\right]},c_{k-1}^{\left[i\right]},t_{k-1}^{\left[i\right]}\right)} \end{array}$$

**Figure 3:**
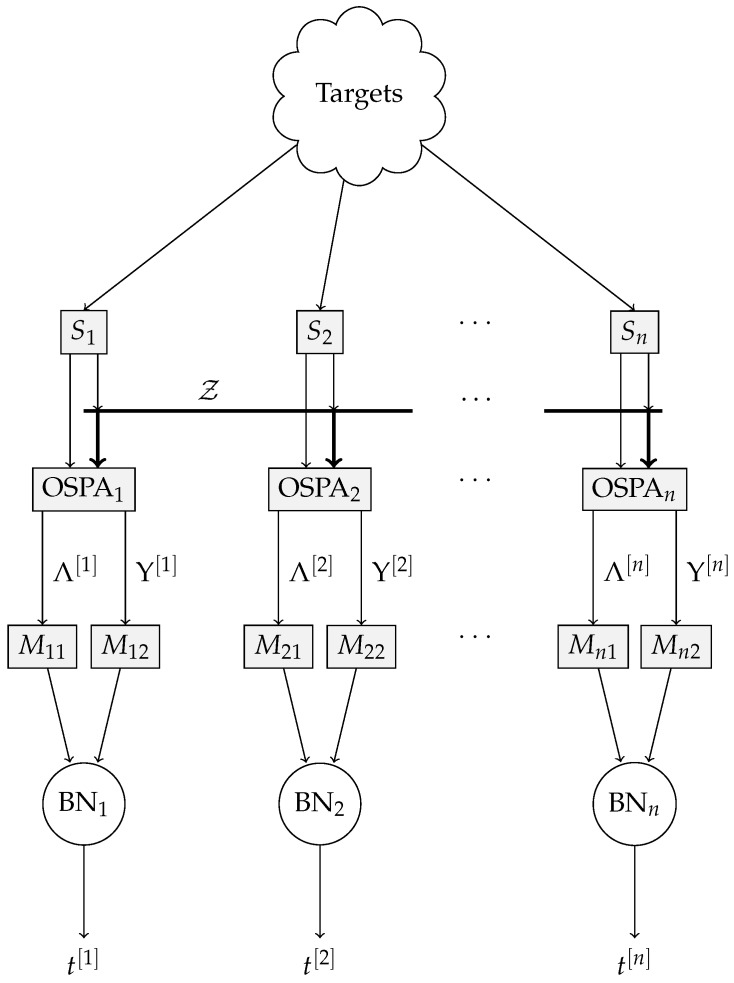
Complete anomaly detection scheme using the Bayesian network approach.

The full anomaly detection system is described in Figure 3. The targets are (generally) visible to all sensors $S_{1},\ldots ,S_{n}$. The components of OSPA Λ and Υ are computed for every sensor, and banks of delays $M_{i1}$ and $M_{i2}$ produce ${\bar{\Lambda }}_{k}^{\left[i\right]}$ and ${\bar{\Upsilon }}_{k}^{\left[i\right]}$, respectively, which are then fed to the individual BNs. The output of each BN is the estimated posterior probability of sensor trustworthiness, $t_{k}^{\left[i\right]}$.

## 4. Results

In order to test the performance of the sensor intrusion methodology developed in this work, we provide results of simulations. To offer a comparison, we have also used a one-class classifier technique from the machine learning domain: support vector machines (SVMs). The main difference between the two approaches is that ours uses specific transition probabilities for each sensor, while the SVM algorithm is trained on pure data and does not include information about the likelihood of the reliability of each sensor. As in the case of our Bayesian approach, the SVM algorithm uses data from the Λ and Υ measurements, as different attacks would affect each of these in a different way. The SVM is trained using a time window of length *l* for Λ and Υ, just as for the Bayesian approach. Because it is a one-class classifier on a fairly high dimensional space, we have used a radial basis function kernel transform [32,33].

We introduce the motion model for the simulations before describing the full environment.

### 4.1. Coordinated Turn Model

The coordinated turn (CT) model is popular for aircraft tracking applications, as it assumes a constant forward velocity and has a state-space representation that uses Cartesian coordinates [34]. A useful property of this model is that it has an exact discrete-time conversion [35,36].

The continuous-time version of the CT model has a state vector

$$x={\left[x\;y\;\dot{x}\;\dot{y}\right]}^{T}$$

with a motion model given by

(26) $$\dot{x}=\left[\begin{array}{cccc} 1 & 0 & 0 & 0\\ 0 & 1 & 0 & 0\\ 0 & 0 & -\omega & 0\\ 0 & 0 & 0 & \omega \end{array}\right]x=A\left(\omega \right)x$$

where ω is the turning rate. Its discrete-time equivalent, with the turning rate added as an extra state, is

(27) $$\begin{array}{cc} x(t+T)= & \left[\begin{array}{c} x+\frac{\dot{x}}{\omega }\sin\left(\omega T\right)+\frac{\dot{y}}{\omega }\left[\cos\left(\omega T\right)-1\right]\\ y+\frac{\dot{x}}{\omega }\left[1-\cos\left(\omega T\right)\right]+\frac{\dot{y}}{\omega }\sin\left(\omega T\right)\\ \dot{x}\cos\left(\omega T\right)-\dot{y}\sin\left(\omega T\right)\\ \dot{x}\sin\left(\omega T\right)+\dot{y}\cos\left(\omega T\right)\\ \omega \end{array}\right]\\ & +\left[\begin{array}{ccc} T^{2}/2 & 0 & 0\\ 0 & T^{2}/2 & 0\\ T & 0 & 0\\ 0 & T & 0\\ 0 & 0 & 1 \end{array}\right]\left[\begin{array}{c} w_{x}\\ w_{y}\\ w_{\omega } \end{array}\right] \end{array}$$

where $w_{x},w_{y}$ and $w_{\omega }$ are zero-mean random variables to model velocity and turning rate uncertainties, and *T* is the sampling period.

### 4.2. Simulated Attacks

In the simulations, we introduced seven attacks. It is important to emphasize that these did not cover the whole spectrum of attacks that could occur, but they did test variations of the basic attacks described in Section 3.1. The simulated attacks were performed on a subset of the sensors, and were as follows:

1. Zero-mean noise introduced.
2. Mobile decoy added.
3. Static decoy inserted.
4. Bias added to the observation of one target.
5. Multiple decoys added.
6. A target surpressed.
7. Bias added to all observations.

**Figure 4:**
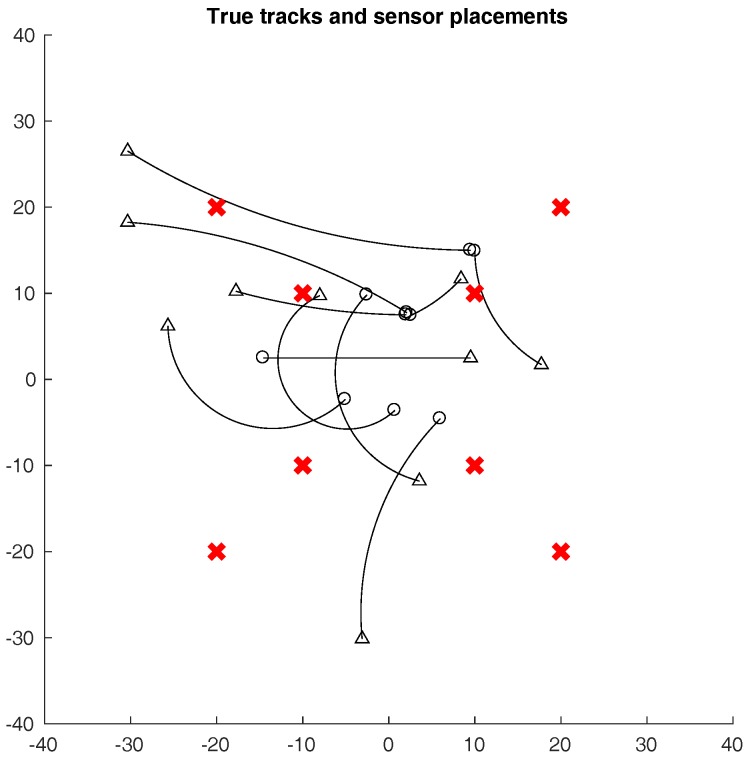
Ground truth of the target tracks. The (∘) markers indicate the start of the target tracks, and their ends are the (▵) markers. The (×) markers indicate the locations of the eight sensors.

For each type of attack, we ran 30 simulations, each with a length of 200 time steps, using the SMC implementation of the PHD filter. The sampling period of the simulations was 1 s. All attacks occurred in the interval 30≤k≤170, with randomly selected start and end times, and also with random duration. For both intrusion detection approaches, we trained the classifiers using a 400 time-step simulation in which the sensors were assumed to be trustworthy. The simulated target tracks, along with the positions of the eight sensors, are shown in Figure 4.

## 5. Discussion

The numerical results report the precision and recall for each classifier, which we have combined by presenting the *F*-score in Table 1. We present two figures per scenario: local and global classification scores. Local indicates that we measured the *F*-score per sensor, so that the overall attack was detected and the responsible sensors were flagged as being compromised. The global *F*-score measured whether an attack was detected by the system, independently from which sensor may have caused it. Hence, the global *F*-score was always expected to be higher.

The attack identifier number from the leftmost column coincides with the seven disruption modalities described before. The numbers in bold font highlight the highest *F*-score in that table row. The column titled “*F*-Score Mode” indicates whether the intrusion detection was computed on a sensor-by-sensor basis (“Per sensor”) or globally (“Global”). In most cases, the “Global” score is significantly higher than the “Per sensor” score. These attacks that were mostly related to the introduction of decoys were particularly well detected. A notable case is attack 6, for which some sensors stopped reporting a subset of the targets. The “Per sensor” *F*-score is low, as the sensors under attack were identified as nominal, and those in normal operation were flagged as compromised. However, the “Global” score is on par with those obtained for other attacks. In this instance, the SVM classifier outperformed the BN on the “Per sensor” *F*-score but not on the “Global” score.

In order to illustrate the effectiveness of both the BN and the SVM approaches under some attacks, we show in detail the results of three simulated attacks. Each attack had a different nature and degree of success. The iterated-corrector SMC–PHD filter had not been altered in any way to change its resilience when presented with the attacks. In these examples, the attack start time was 60 s, with an end time of 150 s.

**Figure 5:**
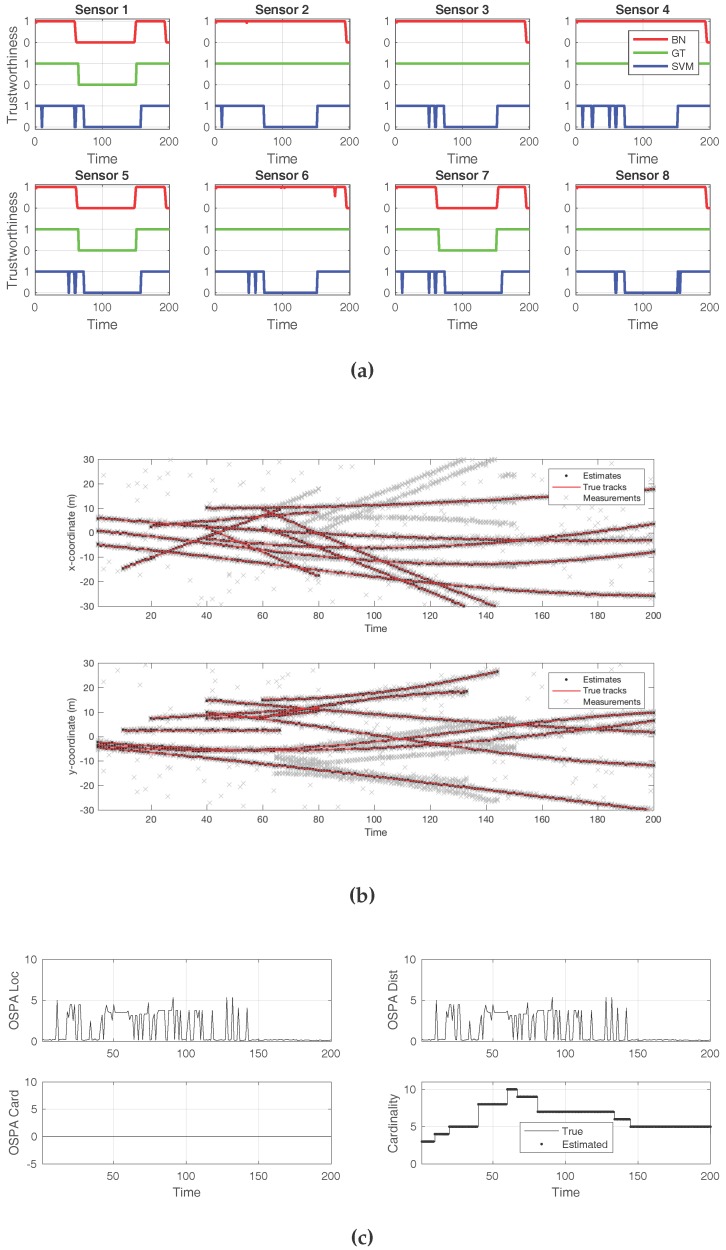
Example of an unsuccessful attack. Several decoys were added to three sensors, out of eight. The cardinality of the detected target set is still correct, even in the presence of the false observations. The Bayesian scheme was able to identify the compromised sensors, while the SVM classifier incurred many false positives. (**a**) Sensor compromise detection by Bayesian network (BN) and support vector machine (SVM), compared to ground truth; (**b**) Tracks detected by the sequential Monte Carlo—probability hypothesis density (SMC—PHD) filter, with every sensor observation displayed; (**c**) Optimal sub-pattern assignment (OSPA) metric, along with its location and cardinality error components. True and estimated cardinalities are also displayed.

First, we show the result of adding several decoys to a minority of sensors, three out of eight. Figure 5 displays plots that convey insight about the effects of the attack. The first plot is Figure 5a, and it compares the ground truth for the attack (green line) to the detection offered by the BN (red line) and to that of the SVM classifier (blue line). In this case, both approaches successfully detected the presence of an attack from a global perspective. However, the SVM classifier flagged every sensor as being under attack. In contrast, the BN correctly identified which sensors were producing the false decoys. Figure 5b shows the tracks detected by the SMC–PHD filter, compared to the true tracks. The gray crosses represent the sensor observations. While there was an abundance of false target reports, along with clutter, this attack was not sufficient to make the filter fail or report the decoys as true targets. The effects of the attack, in terms of the cardinality and localization errors, can be verified in Figure 5c. As this attack was not successful in introducing decoys, the cardinality error was zero, while the localization error remained small.

**Figure 6:**
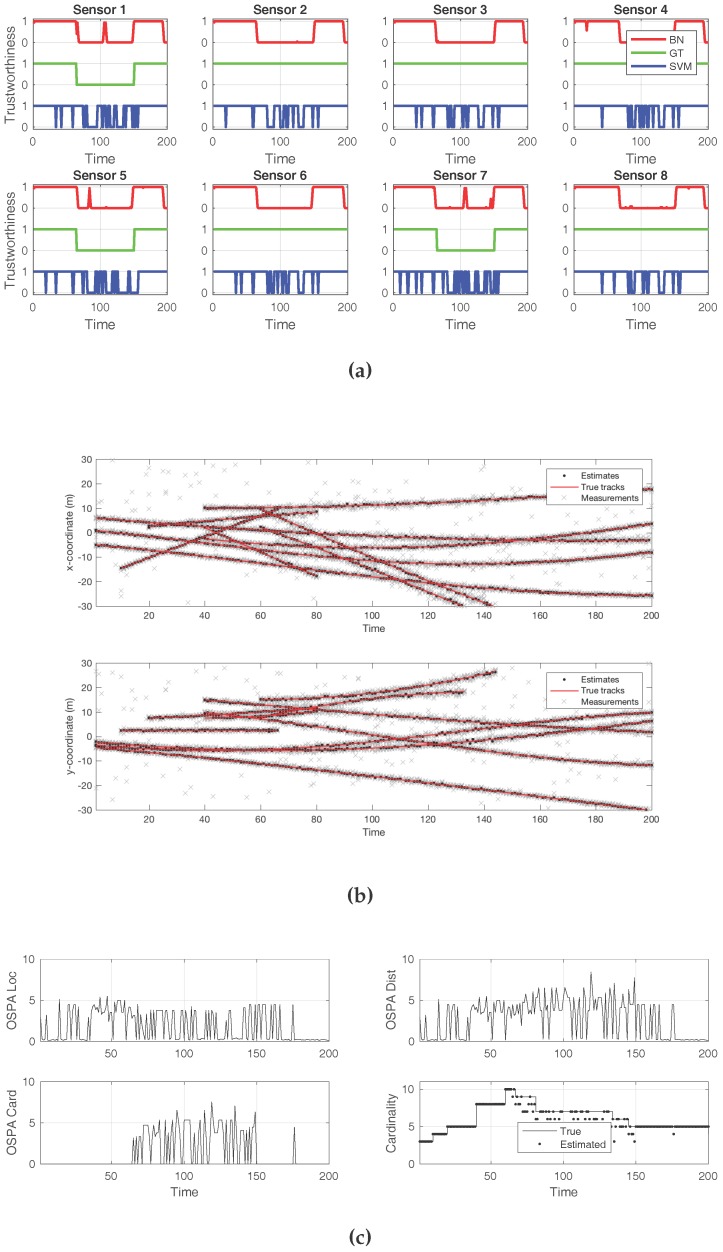
A successful attack. An increased level of white noise was injected to three sensors, out of eight. The cardinality of the detected target set is incorrect for the time of the attack. Both the Bayesian scheme and the SVM classifier flagged many false positives. (**a**) Sensor compromise detection by Bayesian network (BN) and support vector machine (SVM), compared to ground truth; (**b**) Tracks detected by the sequential Monte Carlo—probability hypothesis density (SMC—PHD) filter, with every sensor observation displayed; (**c**) Optimal sub-pattern assignment (OSPA) metric, along with its location and cardinality error components. True and estimated cardinalities are also displayed.

The second case, in Figure 6, exemplifies a successful attack that made the SMC–PHD filter arrive to a wrong cardinality estimate during the times at which the sensors were compromised. Again, three out of the eight total sensors were being attacked. This attack introduced an elevated level of white noise, more than twice what was expected. As Figure 6a depicts, the BN approach incurred several mistakes while identifying individual sensors under attack. The same happened to the SVM classifier. In both cases, even if the compromised sensors were not correctly isolated, the presence of an attack in the overall system was successfully reported. The effects of the attack on the estimated target positions are shown in Figure 6b. Both the localization and the cardinality components of the OSPA error show adverse effects from the attack in Figure 6c. When the estimated cardinality is compared to the ground truth, it can be verified that the noise attack resulted in missed target reports by the filter.

**Figure 7:**
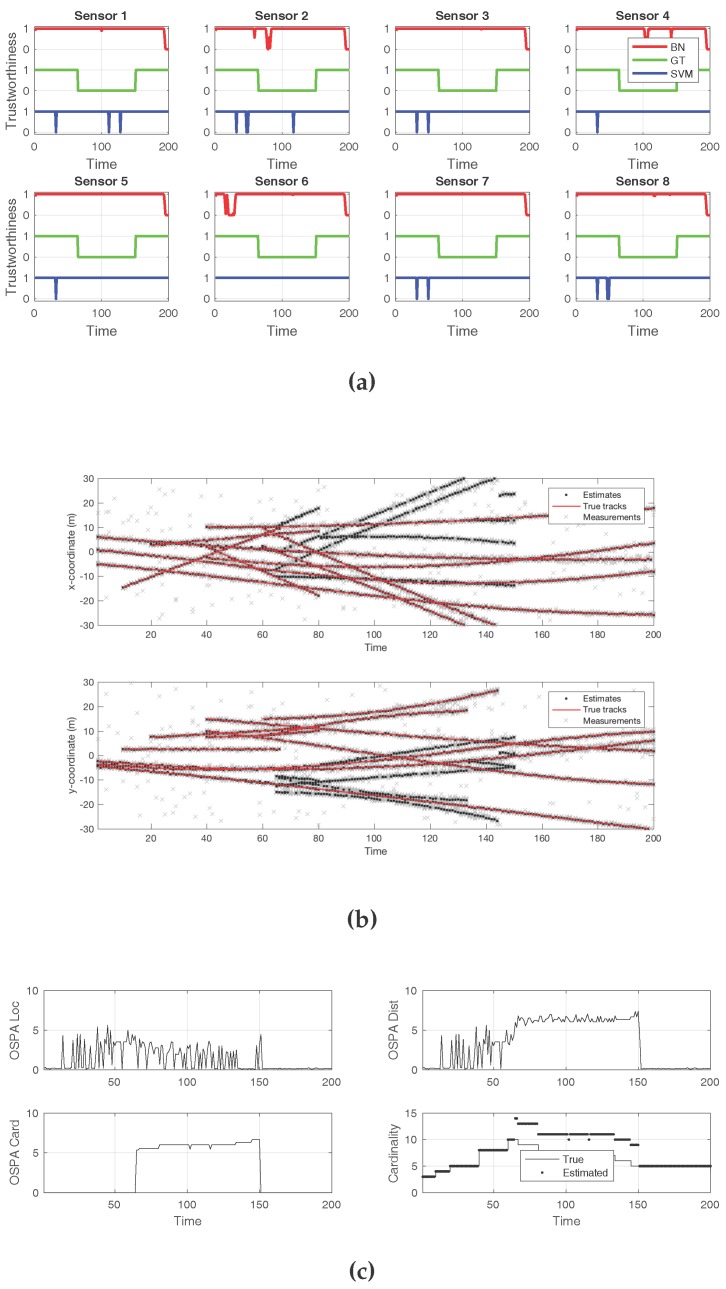
An attack on all sensors. No compromise detection method was able to identify the intrusion. (**a**) Sensor compromise detection by Bayesian network (BN) and support vector machine (SVM), compared to ground truth; (**b**) Tracks detected by the sequential Monte Carlo—probability hypothesis density (SMC—PHD) filter, with every sensor observation displayed; (**c**) Optimal sub-pattern assignment (OSPA) metric, along with its location and cardinality error components. True and estimated cardinalities are also displayed.

The final example scenario, which depicts the effects of an attack on all sensors, is shown in Figure 7. Here, a decoy introduction attack was performed on every sensor, which could not be detected under the schemes discussed in this work. Figure 7a shows that, while the ground truth contained attacks for all sensors, no detection technique reported any compromise. The new, false tracks that resulted from the attack can be seen in Figure 7b. The cardinality error was constantly high during the time of the attacks, as seen in Figure 7c. The comparison between the true and estimated cardinalities shows the increased number of targets during the decoy introduction attack.

## 6. Conclusions

Given the possibility of an intruder taking over a subset of sensors in a target detection and tracking system, we present an algorithm for an effective intrusion detection scheme. We use the principle of a one-class classifier to detect anomalies in the measurements collected by the sensors, without assuming a particular attack form is employed by the intruder. The main tool that enables us to detect the anomalies is the OSPA metric, along with its localization and cardinality error components. While we show that in the single-sensor case, the task of detecting an intrusion can vary from hard to impossible, depending on the type of attack, using multiple sensors opens up the option of forming a consensus set to have some reference behavior with which to compare the performance of individual elements.

As evidenced by our simulation results, the use of a BN per sensor, along with hypothesis testing for the localization and cardinality errors of each sensor with respect to the consensus set, is a powerful tool that identifies the anomalies in a wide variety of situations. Some border cases cannot be handled by this approach if an accurate identification of the culprit sensors is required, but even in these scenarios, an overall intrusion alarm can be raised.

## Figures and Tables

**Table 1:** *F*-scores for different attack classes, on three out of eight sensors.

Attack	*F*-Score Mode	BN Mean	BN Std	BN Best	BN Worst	SVM Mean	SVM Std	SVM Best	SVM Worst
1	Per sensor	0.4447	0.0711	**0.5104**	0.2555	0.3042	0.0720	0.4294	0.1582
1	Global	0.8534	0.0829	**0.9618**	0.6667	0.6190	0.1089	0.7826	0.3214
2	Per sensor	0.8547	0.0501	**0.9174**	0.7494	0.5160	0.0550	0.6008	0.3817
2	Global	0.8919	0.0618	**0.9576**	0.6986	0.8024	0.0944	0.9243	0.5472
3	Per sensor	0.8414	0.0625	**0.9329**	0.6667	0.6434	0.0948	0.7742	0.4393
3	Global	0.8741	0.0656	**0.9600**	0.7126	0.7931	0.0914	0.9200	0.5882
4	Per sensor	0.4843	0.0232	**0.5180**	0.4398	0.4552	0.0489	0.5125	0.3557
4	Global	0.8628	0.0597	**0.9531**	0.7423	0.8162	0.0998	0.9434	0.6133
5	Per sensor	0.8485	0.0547	**0.9300**	0.7550	0.4759	0.0568	0.5684	0.3623
5	Global	0.9051	0.0424	**0.9627**	0.8148	0.8077	0.0871	0.9339	0.6500
6	Per sensor	0.0355	0.0281	0.0724	0.0026	0.2126	0.0636	**0.3232**	0.0778
6	Global	0.8968	0.0449	**0.9565**	0.8000	0.8146	0.0764	0.9381	0.6389
7	Per sensor	0.4812	0.0233	**0.5203**	0.4279	0.4333	0.0383	0.4901	0.3587
7	Global	0.8758	0.0586	**0.9573**	0.7209	0.7842	0.0818	0.9239	0.6250

## Abbreviations

The following abbreviations are used in this manuscript:

BN	Bayesian network
LTI	Linear time-invariant
OSPA	Optimal sub-pattern assignment
PHD	Probability hypothesis density
SVM	Support vector machine
